# Exposure to Fluoride During Pregnancy and Lactation Induces Metabolic Imbalance in Pancreas: A Toxicological Insight Using the Rat Model

**DOI:** 10.3390/ijms26199817

**Published:** 2025-10-09

**Authors:** Marta Skórka-Majewicz, Wojciech Żwierełło, Arleta Drozd, Irena Baranowska-Bosiacka, Donata Simińska, Agata Wszołek, Izabela Gutowska

**Affiliations:** 1 Department of Medical Chemistry, Pomeranian Medical University in Szczecin, 70-111 Szczecin, Poland; marta.skorka.majewicz@pum.edu.pl (M.S.-M.); wojciech.zwierello@pum.edu.pl (W.Ż.); 2 Department of Applied Microbiology and Human Nutrition Physiology, Faculty of Food Sciences and Fisheries, West Pomeranian University of Technology, 70-310 Szczecin, Poland; arleta.drozd@gmail.com; 3 Department of Biochemistry, Pomeranian Medical University in Szczecin, 70-111 Szczecin, Poland; irena.baranowska.bosiacka@pum.edu.pl (I.B.-B.); donata.siminska@pum.edu.pl (D.S.); 4 Institute of Biology, University of Szczecin, 71-415 Szczecin, Poland; agata.wszolek@usz.edu.pl

**Keywords:** fluoride, glucagon, insulin, pancreas, rat

## Abstract

Fluoride is a widespread environmental toxin that disrupts metabolic and endocrine functions, but its impact on pancreatic inflammation and hormone secretion remains unclear. This study examined how chronic fluoride exposure affects pancreatic inflammation and secretory function in rats. Pregnant Wistar rats received sodium fluoride (NaF) at 50 mg/L in drinking water during gestation and lactation. Male offspring continued exposure until 3 months old. Controls received fluoride-free water. Pancreatic tissue and serum were collected. Fluoride levels were measured potentiometrically. Eicosanoids were quantified by SPE and HPLC. Serum insulin, glucagon, and somatostatin were measured by ELISA. Histological and biochemical markers of inflammation and oxidative stress were assessed. Fluoride exposure did not lead to significant accumulation in the pancreas or serum. However, fluoride-exposed rats exhibited a significant decrease in serum insulin and somatostatin concentrations, while glucagon levels remained unchanged. Additionally, the pancreas of fluoride-treated animals showed markedly elevated levels of pro-inflammatory eicosanoids, including prostaglandin E2, leukotrienes A4 and B4, and HETE/HODE derivatives, indicating activation of cyclooxygenase and lipoxygenase pathways. Sustained low-dose fluoride exposure induced pancreatic inflammation and disrupted endocrine homeostasis in rats. These findings suggest that chronic fluoride intake may impair insulin secretion and promote pre-diabetic alterations, warranting further research.

## 1. Introduction

Natural and anthropogenic pollution leads to the continuous release of harmful substances into the environment. As a result, ecosystems become contaminated with heavy metals and toxic elements such as fluoride. It has been demonstrated that chronic exposure to this element causes, among other effects, stimulation of free-radical processes and the initiation and progression of inflammatory processes mediated by cyclooxygenases (COXs) and lipoxygenases (LOXs) in the liver, nephrotoxicity, and changes in the skeletal and muscular systems [1,2,3]. Fluoride ions, due to their high reactivity, can inhibit the activity of many enzymes—including those involved in glucose metabolism (e.g., Krebs cycle enzymes, glycolytic enzymes, and electron transport chain components such as cytochrome oxidase, cytochrome c, succinate dehydrogenase) as well as fatty acid oxidation pathway enzymes [4]. They also inhibit antioxidant enzymes [3], thereby disrupting organ function and exacerbating pathological changes. The effect of fluoride on enzyme activity involves, on one hand, the formation of insoluble complexes with metal cations that form the active centers of many enzymes. On the other hand, negatively charged fluoride ions can bind to functional groups of amino acids surrounding an enzyme’s active site, causing a conformational change and thus enzyme inhibition [5]. Adverse effects of excessive fluoride in the body have been observed in many human organs, including those with endocrine functions [2,6]. Pancreatic hormones play a central regulatory role in glucose metabolism. The pancreas is a glandular organ composed of two types of tissue: exocrine (acinar) and endocrine (islet) tissue. Endocrine cells constitute about 2% of the organ and are located in the islets of Langerhans. These cells synthesize and secrete insulin, glucagon, and somatostatin—the key hormones responsible for proper metabolism not only of carbohydrates but also of fats and proteins [7]. Glucagon and insulin are the two most important hormones responsible for the endocrine control of blood glucose levels, functioning antagonistically to each other [8]. Insulin is an endocrine peptide hormone that binds to plasma membrane receptors on target cells to orchestrate an integrated anabolic response to nutrient availability [9]. In the fed state, high blood glucose triggers insulin release from β-cells in the islets of Langerhans, which in turn initiates the synthesis of glycogen, proteins, and fats. Dietary glucose is utilized by neurons independently of insulin; however, glucose uptake by hepatocytes and myocytes is insulin-dependent [8]. During the postprandial period, glucagon becomes predominant as the body maintains blood glucose by releasing it from hepatic glycogen stores to meet the immediate energy needs of neural tissue. Simultaneously, glycogenolysis occurs in muscle along with the release of energy from triacylglycerol breakdown. During prolonged fasting, there is an intensified breakdown of triacylglycerols stored in adipocytes, and the glycerol released is used for glucose synthesis via gluconeogenesis to supply the whole body. In parallel, gluconeogenesis is supported by amino acids released from protein catabolism, while extensive fatty acid oxidation leads to the synthesis of ketone bodies that serve as an energy source for peripheral tissues and, to a large extent, the brain [10]. The hormone regulating the release of both insulin and glucagon is somatostatin, which also oversees nutrient absorption in the intestines. In addition, somatostatin participates in gastric emptying and limits the secretion of digestive enzymes [11]. Proper hormone secretion by the pancreas depends on its optimal function, which can be disrupted by the negative effects of xenobiotics, including fluorides. The impact of fluorides on pathways modulating the pro- and antioxidant balance has been demonstrated repeatedly [12,13]. Among the compounds involved in these processes are eicosanoids—products of polyunsaturated fatty acid metabolism via cyclooxygenases (COX) and lipoxygenases (LOX) [14]. These compounds can modulate immune activity and initiate or advance inflammatory responses [15], which can lead to the development of type 1 and type 2 diabetes [16]. Studies have shown that COX isoforms [17] and LOX isoforms [18] are differentially involved in controlling the function and destruction of pancreatic β-cells, directly affecting insulin secretion. Additionally, eicosanoids regulate pancreatic blood flow and participate in acute and chronic inflammatory processes in the pancreas [19,20].

Results of studies comparing F levels in maternal and umbilical cord venous blood plasma, as well as intra-uterine studies, prove that F passes through the placenta [21]. Whether and to what extent the placenta can act as a filter and limit transmission of F to fetal circulation remain matters of debate [22,23]. In light of the above, the objective of this study was to examine how exposure to fluoride during pregnancy and lactation in rats (administered in drinking water) influences the development of inflammation in the pancreas and modifies the secretory function of this organ.

## 2. Results

### 2.1. Analysis of Fluoride Ion Concentrations in the Tissues and Serum of Fluoride-Exposed Rats Compared with Controls

There were no statistically significant differences between the groups in the level of fluoride in the serum and in the pancreatic tissue of the rats (Table 1).

### 2.2. Analysis of the Concentration of the Hormones Insulin, Glucagon, and Somatostatin in the Serum of Rats Exposed to Fluoride and Control Group

The analysis of hormone levels showed that exposure to fluoride resulted in a decrease in the amount of insulin (*p* = 0.0005) and somatostatin (*p* = 0.026) in the serum of the fluoride group. No statistically significant differences were observed in glucagon concentration (Table 2).

### 2.3. Analysis of the Fatty Acid Derivatives Concentrations in Pancreas Tissues of Rats Exposed to Fluoride and Control Group

The analysis of fatty acid derivatives showed a statistically significant increase in the concentrations of prostaglandin E2 (*p* = 0.00003), resolvin D1 (*p* = 0.040), leukotriene A4 (*p* = 0.0006), protectin DX (*p* = 0.040), maresin 1 (*p* = 0.006), leukotriene B4 (*p* = 0.001), 13-HODE (*p* = 0.003), 9-HODE (*p* = 0.003), 17-HDHA (*p* = 0.022), and 12-HETE (*p* = 0.022) in the pancreas tissue of the fluoride group compared to control.

**Figure 1:**
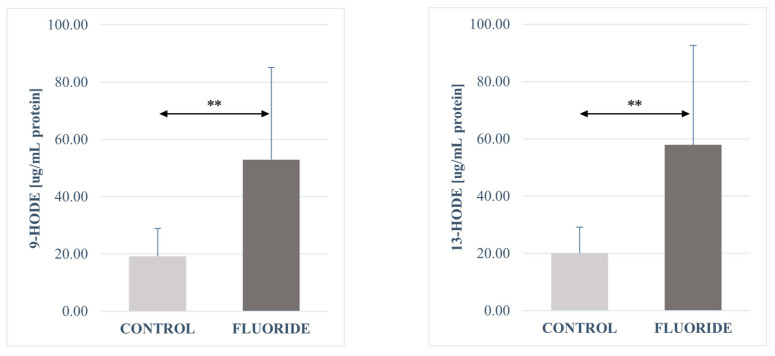
Concentrations of 9-HODE and 13-HODE in pancreatic tissue (μg/mg protein). Data are mean ± SD, n = 6. Significant differences observed between groups: **—*p* < 0.0001.

**Figure 2:**
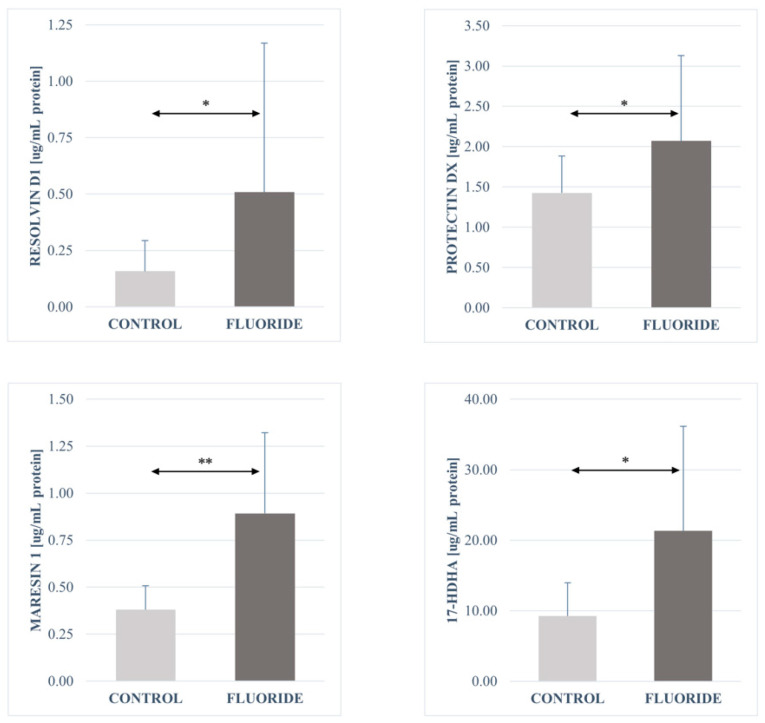
Concentrations of Resolvin D1, Protectin DX, Maresin 1 and 17-HDHA in pancreatic tissue (μg/mg protein). Data are mean ± SD, n = 6. Significant differences observed between groups: *—*p* < 0.05, **—*p* < 0.0001.

**Figure 3:**
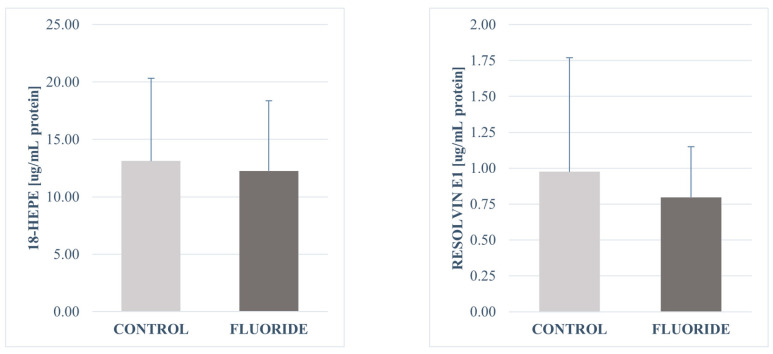
Concentrations of Resolvin E1 and 18-HEPE in pancreatic tissue (μg/mg protein). Data are mean ± SD, n = 6.

**Figure 4:**
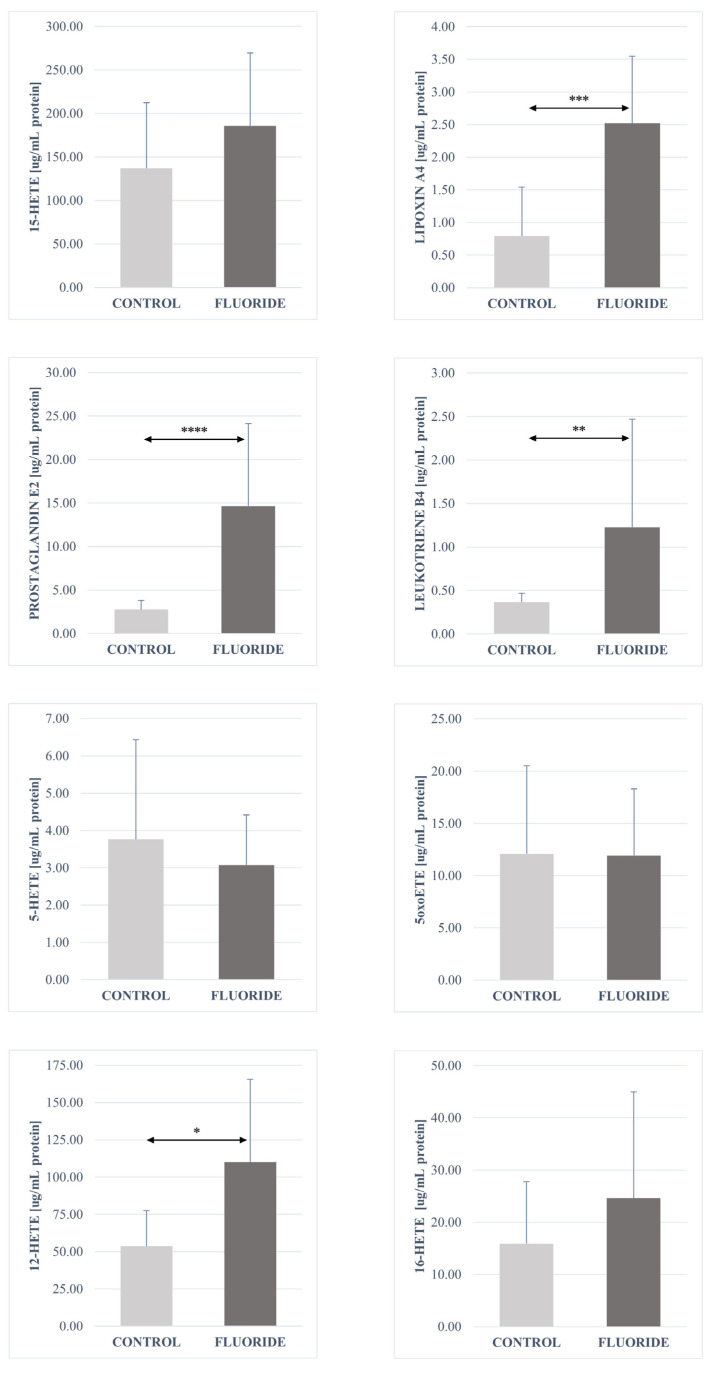
Concentrations of 15-HETE, Lipoxin A4, Leukotrien A4, Prostaglandin E2, 12-HETE and 16-HETE in pancreatic tissue (μg/mg protein). Data are mean ± SD, n = 6. Significant differences observed between groups: *—*p* < 0.05, **—*p* < 0.0001, ***—*p* < 0.00001, ****—*p* < 0.00005.

No significant differences were found in the concentrations of the other determined compounds. The concentrations of 16-HETE and 15-HETE in the fluoride-exposed group increased slightly. A decrease in the amount was observed for resolvin E1 and 18-HEPE. The same level of 5-oxo-ETE and 5-HETE was observed in both groups (Table 3, Figure 1, Figure 2, Figure 3 and Figure 4).

## 3. Discussion

There are only a few reports on the effects of fluoride on the human endocrine system. Most available studies have focused on fluoride’s toxicity to the testes, ovaries, and thyroid gland. Some publications suggest that NaF exposure can significantly affect insulin levels and glucose-regulating proteins. However, data regarding the influence of fluoride on inflammatory processes in the pancreas are lacking. To date, no studies have examined the interaction of fluoride with the activation of cyclooxygenases or lipoxygenases in the pancreas, even though disturbances of the endocrine pancreas can lead to serious pathologies.

### 3.1. Fluoride Accumulation in the Rat Body

Our measurements of fluoride in serum and pancreatic tissue indicate that neither serum nor pancreatic tissue serves as a site of long-term fluoride accumulation. The primary tissue for fluoride deposition is bone, where fluoride is incorporated into the hydroxyapatite structure (forming fluorohydroxyapatite or fluoroapatite). It is reported that only about 1% of absorbed fluoride accumulates in soft tissues, which aligns with the lack of statistically significant differences we observed in fluoride levels in pancreatic tissue versus serum. Additionally, tissue fluoride levels decline within 3–6 h after absorption, and most of the remaining fluoride is excreted in urine within 24 h [24]. Some studies suggest that certain soft tissues can be more susceptible to fluoride accumulation; for example, fluoride has been found to accumulate in the brain, kidneys, liver, and lungs to a greater extent than other soft tissues [3,10,25].

### 3.2. Concentration of Fatty Acid Derivatives in the Pancreas

Arachidonic acid and its metabolites can have a significant impact not only on the development of inflammatory processes within the pancreas but also on its secretory functions [26]. Eicosanoids and their derivatives participate not only in the regulation of physiological functions but also mediate inflammatory responses in various tissues of the body. Acute and chronic inflammatory processes within the pancreas underlie dysfunctions of this organ, including abnormalities in the production of hormones such as glucagon, insulin, and somatostatin [26].

The main cause of the development of type 1 or type 2 diabetes is the loss or impaired functioning of pancreatic beta cells [27]. Overexpression of COX-2 in pancreatic cells results in an increased frequency of acute inflammatory episodes of this organ [28]. Among lipoxygenase-derived metabolites, the isoforms 12-HETE and 15-HETE play the most important role in pathological processes within the pancreas [29,30]. In in vitro studies, it was observed that glucose-induced insulin secretion by isolated rat pancreatic islets is inhibited by 15-HETE and 12-HETE. In contrast, 5-HETE induces insulin secretion in medium with low glucose concentrations [31]. The results of these studies were confirmed in vivo. An increase in the concentrations of 15-HETE, leukotriene B4, and leukotriene C4 caused inhibition of insulin release, while 5-HETE and 12-HETE did not change secretory functions in rats [29,32].

Researchers are also interested in LOX enzymes, particularly 12-LOX and 15-LOX [30]. 12-Lipoxygenase is a key isoform present in human pancreatic islets and is primarily responsible for the production of 12-HETE from arachidonic acid [33,34]. The derivatives probably play an important role in the process of pancreatic beta-cell destruction and the pathogenesis of both type 1 and type 2 diabetes [35]. Inhibition of lipoxygenase activity by its inhibitor NDGA (Nordihydroguaiaretic acid, 1,4-Bis(3,4-dihydroxyphenyl)-2,3-dimethylbutane,4,4′-(2,3-dimethyltetramethylene)dipyrocatechol, Masoprocol) results in suppression of cytokine-induced beta-cell destruction in rats [36]. Tunaru et al. observed that 20-HETE can act as an autocrine positive feedback stimulator of insulin, and additionally, pancreatic islets release it in a glucose-dependent manner. Furthermore, it was observed that this mechanism mediated by 20-HETE is impaired in patients and animals diagnosed with diabetes [37,38].

The action of prostaglandins on tissues depends on their concentration and the type of inflammatory process ongoing in the body. Chronic inflammatory states involving prostaglandins can lead to a decrease in the number of pancreatic beta cells due to suppression of their regenerative capacity [39]. It has been shown that prostaglandin E inhibits insulin secretion, and some inhibitors of PG synthesis enable glucose-induced insulin release via a negative feedback loop in beta cells both in healthy individuals and in patients with type II diabetes [32]. Prostaglandin E2 can contribute to beta-cell damage and activate pro-inflammatory processes through ERK, NFκB, and JNK/STAT3 pathways [16,40]. An exogenous increase in PGE2 synthesis inhibited insulin secretion via IL-1beta, whereas administration of selective COX-2 inhibitors reversed this effect in rat pancreatic cells [41].

Srivastava et al. observed in patients with type 2 diabetes increased expression of the EP3 receptor (for prostaglandin E2), which led to beta-cell failure. The researchers concluded that reducing the expression of the mEP3γ isoform of this receptor could contribute to protecting cells against apoptosis [42]. High concentrations of COX pathway derivatives such as PGE2, PGD2, and TXB2 were also observed in acute and chronic pancreatitis in animal models [29,43,44]. Additionally, PGE2 decreases tissue insulin sensitivity, contributing to hyperglycemia [45,46]. Prostaglandins may also be involved in regulating glucagon secretion by modulating the hormonal response of pancreatic islets [47]. However, some studies indicate protective functions of prostaglandins against acute pancreatic injury [48,49].

Vitamin C supplementation in individuals with hyperglycemia and hypertension caused an increase in the concentration of PGE1, PGI2, and LXA4, exerting cytoprotective effects and protecting pancreatic beta cells [50]. The results of our study indicate a significant increase in PGE2 concentration after fluoride intoxication in the rat pancreas. Simultaneously, an increase in the concentration was also observed for TXB2 and LXA4 compared to the control group. The obtained results may indicate chronic pancreatic inflammation in animals subjected to fluoride compound intoxication, since chronic inflammation of this organ is characterized by elevated levels of prostaglandin (PG), thromboxane (TX), and platelet-activating factor (PAF), in contrast to acute pancreatitis models, where leukotriene B4 increases, TXB2 decreases, and PGE2 concentration remains unchanged [43,51,52,53].

It appears that n-3 PUFA fatty acids may play protective roles against diabetes development. This group includes derivatives of ALA, EPA, and DHA. They exert beneficial effects by lowering insulin levels and preventing beta-cell damage [54]. It has been shown that DHA promotes glucose-dependent insulin secretion in rat pancreatic beta cells. Additionally, its action is based on increasing intracellular calcium ion concentration and inhibiting voltage-dependent K+ channels [55]. Another study showed that docosahexaenoic acid inhibits cytokine expression and reduces reactive oxygen species in isolated rat pancreatic stellate cells [56].

It has been observed that resolvin D1 in combination with streptozotocin can alleviate type 1 diabetes by lowering TNF-α, IL-6, and BDNF plasma concentrations and downregulating gene expression in the Bcl2/Bax/COX-1/COX-2/PPAR-γ pathway in rat pancreas. Additionally, it exhibits anti-apoptotic, antioxidant, and anti-inflammatory effects [57,58]. In turn, resolvin E1 exerts anti-apoptotic action by inhibiting pro-inflammatory markers and lowering the ADP/ATP ratio in an in vitro experiment on human pancreatic islets [59]. Furthermore, EPA and DHA acids, by displacing arachidonic acid from cell membrane lipids, increase LXA4 production, which has anti-inflammatory properties. The author suggests that resolvins, protectins, maresins, and lipoxins, besides their anti-inflammatory actions, may exert cytoprotective effects by enhancing beta-cell proliferation and inducing differentiation of pancreatic stem cells, leading to increased insulin production and reduced hyperglycemia [60].

Free radicals and oxidative stress play a significant role in the activation of inflammatory processes by HETE/HODE, and both processes are strongly induced by fluoride ions, as proven in numerous studies [61]. It has been shown that pancreatic beta cells are highly sensitive to these mechanisms. Reactive oxygen species cause calcium influx into the cytoplasm by activating phospholipase C (PLC) and phosphorylating inositol triphosphate–sensitive calcium channels. This process results in calcium release into the cytosol, leading to the induction of various intracellular signals regulated by this element, including the activation of prostanoids derived from arachidonic acid [61,62]. In patients with type 1 and type 2 diabetes, elevated markers of oxidative stress such as MDA have been observed, along with increased levels of lipid peroxidation markers like prostaglandin F isomers. Available literature confirms that activation of fatty acid derivative synthesis pathways can be caused by ROS both in vitro on cell lines and in vivo in laboratory animals [61]. Gutowska et al. demonstrated that increased concentrations of 15- and 12-HETE and 9- and 13-HODE were accompanied by superoxide anion activation in studies using fluoride compounds as the toxic factor [52].

### 3.3. Influence of Fluoride on Pancreatic Hormone Concentrations

The conducted studies demonstrated that intoxication of rats with sodium fluoride causes a significant decrease in insulin concentration (by 19.91%) and somatostatin (by 11.49%) compared to the control group. In the case of glucagon, a statistically insignificant increase in its serum concentration (by 10.44%) was recorded.

The pancreas is an organ essential for metabolic control. Maintaining homeostasis in the concentrations of the hormones it produces—insulin, glucagon, and somatostatin—enables the effective functioning of the entire organism. Hyperinsulinemia is often observed in pre-diabetic states [63]. Tissue insulin resistance and reduced glucose transport favor increased production of this hormone by the pancreas. Chronic disturbances in insulin release and insulin resistance contribute to pancreatic insufficiency, leading to the loss of beta-cell functionality and insulin deficiency [64]. In the literature, there are few reports on the influence of fluoride on the pancreas and its endocrine functions. The available publications mainly concern insulin and glucose regulation processes.

Acute and chronic exposure to fluoride can result in disturbances of glucose homeostasis in the body. In the study by Lupo et al., an increase in plasma insulin concentration was correlated with higher doses of ingested fluoride, yet this had no effect on plasma glycemia. This effect was noticeable at lower concentrations of sodium fluoride (from 5 to 20 mmol/L) [65]. In a year-long study conducted on 25 Wistar rats exposed to NaF at a concentration of 100 mg/L, an increase in insulin level and a decrease in serum glucagon concentration were recorded, and immunohistochemical analysis of pancreatic islets revealed an increased insulin-positive area [66]. Rigalli et al. observed inhibition of glucose-stimulated insulin secretion from Langerhans islets. Abnormal glucose tolerance tests were also demonstrated in animals from the study group [67]. Another experiment using the same sodium fluoride concentrations showed that as the amount of this element in drinking water increased, there was a transient decrease in insulin secretion by pancreatic islets [68]. These findings are confirmed by the work of García-Montalvo et al., indicating that high fluoride concentrations in water promote reduced insulin secretion from beta cells and lower mRNA expression for insulin despite glucose stimulation [69].

The decrease in insulin levels after fluoride intoxication is noticeable in short-term study designs. After a single oral dose of fluoride (40 mmol/100 g body weight), glycemia values and insulin concentrations return to normal within 5 h [70]. In contrast, chronic fluoride intake favored decreased insulin secretion, the development of insulin resistance, and hyperglycemia. It was also observed that fluorides at various concentrations can cause negative histological changes in pancreatic cells, particularly involving changes in the appearance of mitochondria and the endoplasmic reticulum [71,72]. Studies conducted on humans diagnosed with fluorosis have shown decreased blood insulin concentrations, impaired glucose tolerance, and high fasting glucose levels. The above studies suggest that chronic fluoride exposure may cause glucose intolerance and abnormalities in insulin secretion [73].

Researchers suggest that somatostatin may also have an insulin-like effect. Octreotide is a pharmacological agent that mimics the natural action of somatostatin. It was observed that its administration in therapy limits the inflammatory response, mainly by reducing the secretion of pro-inflammatory cytokines and TNF-α. Additionally, it lowers MDA levels and increases total antioxidant capacity (TAC) [73]. In contrast to insulin and somatostatin, glucagon most likely affects the reduction in the body’s antioxidant capacity. Studies suggest that this hormone completely inhibits the expression of GST protein, while insulin induces expression of the gene for this enzyme. Similar reductions in the amounts and activities of antioxidant enzymes were observed when assessing the influence of glucagon on the synthesis of glutathione (GSH), which exerts a protective effect on protein thiol groups [74].

In a study conducted by Trevizol et al. on 42 female NOD mice, metabolic changes in pancreatic islets exposed to 10 mg F/L were assessed. After 14 weeks of fluoride administration in drinking water, the authors did not observe statistically significant differences in pancreatic inflammatory infiltrates or in the mean percentages of cells stained for insulin, glucagon, and acetylated histone H3 [75]. An earlier study by the same authors showed decreased serum glucose levels without any effect on insulin levels. The authors suggest that fluoridation of drinking water may play an important role not only in preventing dental caries but also diabetes, through the reduction in phosphoenolpyruvate carboxykinase (PEPCK), an enzyme involved in increasing glucose uptake [76].

Sodium fluoride at a dose of 500 ppm administered to rats in distilled water for 60 days increased pancreatic amylase and lipase activity compared to the group that received only distilled water (control). Additionally, increased levels of interleukin-6, interleukin-10, and TNF-α in the pancreas were demonstrated compared to the control group. In animals receiving NaF, CAT and SOD activity and GSH levels—markers of oxidative stress—decreased, and an increase in the concentration of caspase-3, the Bax/Bcl-2 ratio, and hydroxyproline was observed, which may indicate progressing pancreatic fibrosis [77]. Similar processes were studied by Aslan et al., in an experiment in which rats were fed a standard diet supplemented with fluoride at a concentration of 50 and 100 mg/kg body weight for 8 weeks. The studies showed that pancreatic cells of these animals were characterized by reduced glutathione levels, catalase, Bax protein, and caspase-3 and -6. Increased activity was observed for Bcl-2 protein, TNF-α, and NF-κB [78].

Thakur et al., in their review paper on the role of fluoride in diabetes development, suggest that excess dietary fluoride may affect disturbances of glucose homeostasis and increased insulin resistance. It was noted that fluoride exposure decreases insulin and glucagon levels and alters the secretion of these hormones from the islets of Langerhans. Literature analysis suggested decreased antioxidant enzyme activity (CAT, GPx, SOD), increased levels of oxidative stress markers (lipid peroxidation, ROS, MDA), inflammatory markers (TNF-α, IL-6), and morphological changes in pancreatic tissues [79]. Similar conclusions were drawn after conducting a meta-analysis concerning changes induced by fluoride in the pancreas of mammals. Rana et al., after analyzing 51 publications containing keywords such as “fluoride,” “pancreas,” “toxicity,” and “insulin,” indicate that fluoride significantly affects not only changes in the morphology of pancreatic cells but also disrupts hormonal regulation, inhibits digestive enzyme activity, and induces oxidative stress and inflammation [80].

The results presented in this study confirm the scarce literature data, particularly those describing the impact of fluoride ions on disturbances in insulin secretion and a decrease in serum insulin concentration. The slight increase in glucagon may be a consequence of the reduction in insulin and somatostatin levels, indicating an initiated process leading to the emergence of a pre-diabetic state, which, along with elevated eicosanoid concentrations, could result in damage to pancreatic beta cells. The consequence of this process may be hyperinsulinemia combined with insulin resistance and reduced insulin secretion. However, to confirm this hypothesis, further studies focused on glycemia disturbances in long-term experiments should be undertaken.

## 4. Materials and Methods

### 4.1. Fluoride Toxicity Model In Vivo

**Figure 5:**
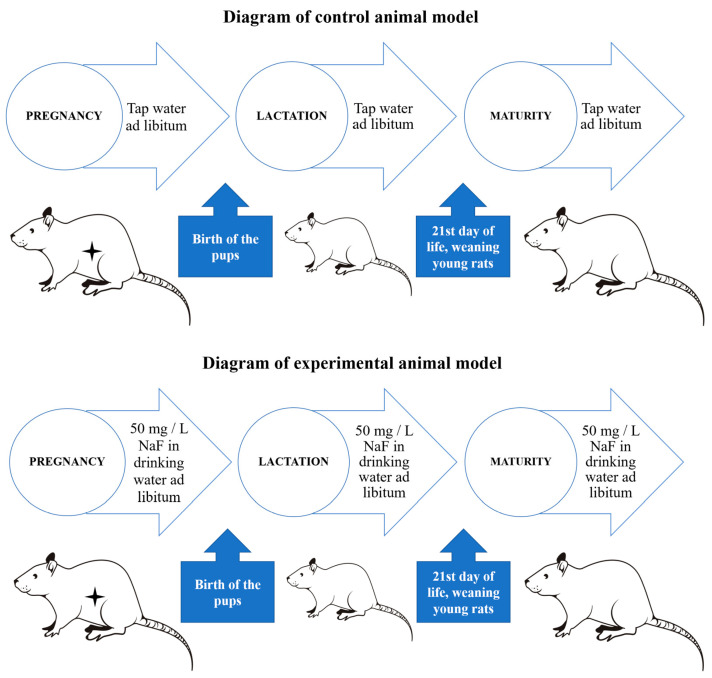
Diagram of the experimental and control animal model.

The animal experiment was carried out in accordance with the guidelines and with the approval of the Bioethics Committee of the Pomeranian Medical University in Szczecin (Decision No. 32, 22 May 2015). As shown in Figure 5, the study was conducted on Wistar strain rats. Adult females were placed in the cages with sexually mature males for 7 days, after which the pregnant females were separated and randomly divided into two groups—test and control. Pregnant female rats (n = 6) in the fluoride-exposed group received sodium fluoride (NaF) in their drinking water at a concentration of 50 mg/L ad libitum throughout pregnancy and the nursing period. The offspring (n = 6) were separated from the mothers on postnatal day 26 and housed in separate cages by sex. Subsequent procedures were conducted only on male offspring to avoid the confounding effects of female hormonal cycles. These male rats continued to receive NaF solution (50 mg/L) until reaching full maturity (3 months of age). The control group (n = 6) was maintained under the same conditions except the animals received fluoride-free tap water ad libitum. At 3 months of age, all animals were euthanized. The rats were anesthetized by intraperitoneal administration of ketamine (80 mg/kg body weight) and xylazine (10 mg/kg). Under deep anesthesia, an incision was made through the skin and abdominal muscle wall, the sternum was cut, and the thoracic cavity was opened. Blood was collected from the heart (cardiac puncture), after which the heart was incised to ensure euthanasia. The blood was immediately centrifuged to obtain serum, which was securely stored until analysis. The excised pancreases were immediately flash-frozen in liquid nitrogen and stored at −80 °C until further analyses. The administered dose of 50 mg/L NaF in rats is equivalent to an approximately 10 mg/L fluoride concentration in human drinking water [81]. A rat consumes roughly 30–50 mL of water per day, corresponding to about 1.5–2.5 mg of fluoride intake per day. By comparison, the recommended maximum daily fluoride intake for an adult human is about 3–4 mg (depending on sex). Studies have shown that in a 70 kg adult human, symptoms of fluorosis occur at daily fluoride intakes exceeding 10 mg [82].

### 4.2. Determination of the Concentration of Fluoride Ions in Tissue and Serum Homogenates Using the Potentiometric Method

A potentiometric method with a fluoride ion-selective electrode (9609BNWP, Thermo Fisher Scientific, Warsaw, Poland) was used to measure fluoride ion concentrations in serum and pancreatic tissue. Prior to measurement, samples were prepared as follows:

**Tissues:** Pancreatic tissue samples were dried at 105 °C and ground to a powder in a ceramic mortar. A 10 mg portion of dried, homogenized tissue was weighed and mixed with 1 mL of 2 M HClO_4_. The mixture was incubated with shaking in a thermomixer for 60 min at 90 °C. After this digestion, 0.5 mL of the sample was taken and combined with 2.5 mL of TISAB II buffer (Sigma-Aldrich, Warsaw, Poland) and 2 mL of sodium citrate solution (Chempur, Szczecin, Poland) to bind any ions that could interfere with the fluoride measurement.

**Serum:** A 0.5 mL volume of serum was mixed with 0.5 mL of TISAB III buffer (Merck Millipore, Warsaw, Poland).

Each prepared sample was placed on a magnetic stirrer, and the electrode potential was recorded for 5 min. Then, a fluoride standard (corresponding to the measured potential, in the range of 1 × 10^−4^ to 2 × 10^−5^ M) was added, and the measurement was recorded for an additional 5 min. The two readings were used to calculate the fluoride ion concentration in the sample based on the difference in electrode potential, according to the manufacturer’s instructions. Each sample was measured in duplicate, and the results were averaged.

### 4.3. Determination of Fatty Acid Derivative Concentrations

**Tissue sample preparation:** Using C18 solid-phase extraction (SPE) columns (Agilent Technologies, Cheadle, UK), we extracted the following fatty acid derivatives from pancreatic tissue samples: resolvin E1, resolvin D1, prostaglandin E2, leukotriene A4 (LTX A4), dihydroxy-docosahexaenoic acid (DiHDHA, also known as protectin DX), maresin 1, leukotriene B4, 18-HEPE, 16-HETE, 13-HODE, 9-HODE, 15-HETE, 17-HDHA, 12-HETE, 5-oxo-ETE, and 5-HETE. Pancreatic tissues were first pulverized in liquid nitrogen using a freezer mill, then further homogenized with a blade homogenizer (for 3 s in 500 μL of ice-cold PBS). The homogenates were centrifuged at 7000 rpm for 10 min at 4 °C. We added 1 mL of ice-cold 100% acetonitrile (Sigma-Aldrich, Warsaw, Poland) and 50 μL of internal standard PGB_2_ (10 ng/mL) to the supernatant. After incubation for 10 min at –20 °C, the samples were centrifuged again (20 min at 3000× *g*, 4 °C). The resulting supernatant was acidified by adding 4.5 mL of 1 mM HCl and adjusting the mixture to pH 3 with 1 M HCl (Sigma-Aldrich, Poland). The prepared samples were then subjected to SPE on Bakerbond C18 columns (J.T. Baker, Radnor, PA, USA) according to the manufacturer’s protocol.

**HPLC analysis:** The concentrations of LOX- and COX-derived fatty acid metabolites were analyzed using an Agilent 1260 series high-performance liquid chromatography system consisting of a G1379B degasser, G1312B binary pump, G1316A thermostatted column oven, and G1315C diode-array detector (DAD VL+). System control, data acquisition, and analysis were performed with Agilent ChemStation software (version B.04.03, Agilent Technologies, Cheadle, UK). Chromatographic separation was achieved on a Thermo Scientific Hypersil BDS C18 column (100 mm × 4.6 mm, 3 μm particle size; cat. no. 28103-104630) with the column oven set to 25 °C. The mobile phase consisted of a gradient of solvent A (methanol/water/acetic acid, 50/50/0.1, *v*/*v*/*v*) and solvent B (methanol/water/acetic acid, 100/0/0.1, *v*/*v*/*v*), both prepared with HPLC-grade methanol and glacial acetic acid (Sigma-AldrichWarsaw, Poland). The gradient program was: 30% B at 0.0 min, linear increase to 80% B by 20.0 min, then 98% B from 20.1 min to 23.9 min, and a return to 30% B from 24.0 min to 28.0 min. The flow rate was 1.0 mL/min. Quantification of each analyte was based on integrated peak areas using an internal standard calibration curve.

### 4.4. Measurement of Insulin, Glucagon, and Somatostatin in Serum

Serum concentrations of insulin, glucagon, and somatostatin were measured using ELISA kits (Wuhan Fine Biotech Co., Wuhan, China) and spectrophotometric detection. The specific ELISA kits used were FineTest ER1113 for insulin, ER0488 for glucagon, and ER0331 for somatostatin. All samples were prepared and assayed according to the manufacturer’s instructions. The absorbance was measured at 450 nm using a microplate reader.

### 4.5. Measurement of Protein Concentration

Pancreatic tissue protein content was determined using the Micro BCA Protein Assay Kit (Thermo Scientific, Waltham, MA, USA). In preparation, 2 μL of 50% NaOH was added to 23 μL of each tissue homogenate. The mixture was heated at 60 °C in a thermomixer for 45 min. Then, 180 μL of double-distilled water was added, and the samples were vortexed. Each sample (20 μL) was loaded into a well of a 96-well plate. Next, 130 μL of distilled water and 150 μL of Working Reagent were added to each well (per kit protocol). The plate was incubated for 2 h at 37 °C. Absorbance was then measured at 562 nm with a plate reader, and protein concentrations were calculated from a BSA standard curve.

### 4.6. Statistical Analysis

Statistical analysis was performed using Statistica 13.1 (StatSoft, Krakow, Poland). For each measured parameter, the mean and standard deviation (SD) were calculated. The Shapiro–Wilk test was applied to assess normality of data distribution. Because the distribution deviated from normal, non-parametric tests were used. The Mann–Whitney U test was employed to evaluate differences between groups. Differences were considered statistically significant at *p* ≤ 0.05. Significance levels are indicated as follows: *—*p* < 0.05, **—*p* < 0.0001, ***—*p* < 0.00001, ****—*p* < 0.00005; ns = not significant.

## 5. Conclusions

To our knowledge, no other studies have examined the impact of fluoride compounds on pancreatic HETE/HODE derivative levels and hormone secretion. Despite the lack of direct research on this topic, we can draw certain inferences based on related pathways and mechanisms. Fluoride appears to stimulate the synthesis of fatty acid derivatives through multiple routes. One mechanism is likely via signaling molecules such as reactive oxygen species (ROS), which is a well-documented direct effect of fluoride toxicity. Fluoride also raises levels of pro-inflammatory mediators (e.g., various interleukins, TGF-β, IFN-γ, and NF-κB). These factors collectively activate the production of pro-inflammatory lipid mediators, contributing to tissue inflammation. On the other hand, derivatives of the omega-3 fatty acids EPA and DHA exert anti-inflammatory and cytoprotective effects, helping to resolve inflammation and repair fluoride-induced cellular damage. The observed shifts in levels of pro- and anti-inflammatory mediators derived from linoleic, arachidonic, eicosapentaenoic (EPA), and docosahexaenoic (DHA) acids support the hypothesis that fluoride promotes inflammation in soft tissues, even though these tissues have a limited capacity to accumulate fluoride. Although literature on fluoride’s effects on the endocrine system is scarce and many underlying mechanisms remain unknown, it can be postulated that chronically administered, even physiologically low doses of fluoride may adversely affect endocrine homeostasis and drive pathophysiological processes in endocrine organs. The pancreas may not significantly accumulate fluoride over time; however, as our results demonstrate, it is nonetheless sensitive to fluoride’s action, which leads to significant alterations w its secretory function.

## Figures and Tables

**Table 1:** Fluoride levels in serum and pancreatic tissue of control and fluoride-exposed rats (mean ± SD; n = 6). No significant differences were observed between groups (ns, not significant).

Fluoride Concentration	Control		Fluoride		*p*
	Mean	SD	Mean	SD	
**Serum [mg/L]**	0.177	0.041	0.191	0.105	ns
**Pancreas [mg/kg d.w.]**	21.382	5.371	21.343	3.062	ns

**Table 2:** Serum concentrations of insulin, glucagon, and somatostatin in control and fluoride-exposed rats. Data are presented as mean ± SD; n = 6. Significant differences observed between groups: *—*p* < 0.01, ***—*p* < 0.0001.

Hormone Concentration in Serum [pg/mL]	Control Group		Fluoride Group		*p*
	Mean	SD	Mean	SD	
**Insulin**	197.002	63.168	157.781	64.311	***
**Glucagon**	150.527	62.936	166.247	92.166	ns
**Somatostatin**	1539.312	1090.287	1362.465	1062.393	*

**Table 3:** Concentrations of fatty acid derivatives in pancreatic tissue (μg/mg protein). Data are mean ± SD, n = 6. Significant differences observed between groups: *—*p* < 0.05, **—*p* < 0.0001, ***—*p* < 0.00001, ****—*p* < 0.00005.

Eicosanoids in Pancreas [µg/mg Protein]	Control		Fluoride		*p*
	Mean	SD	Mean	SD	
Resolvin E1	0.9756	0.7943	0.7975	0.35186	-
Prostaglandin E2	2.7551	1.0522	14.6683	9.44807	****
Resolvin D1	0.1577	0.13661	0.5086	0.65992	*
LTX A4	0.7941	0.74794	2.5184	1.03137	***
Protectin DX	1.425	0.4579	2.0717	1.05778	*
Maresin 1	0.3799	0.12804	0.892	0.42921	**
Leukotriene B4	0.3671	0.10222	1.227	1.24162	**
18-HEPE	13.1204	7.19018	12.2518	6.12348	-
16-HETE	15.9062	11.89287	24.6099	20.34443	-
13-HODE	20.0553	9.06033	57.8731	34.67874	**
9-HODE	19.1437	9.78235	52.9248	32.2025	**
15-HETE	136.9282	75.35555	185.5246	83.83571	-
17-HDHA	9.2439	4.72145	21.3315	14.84319	*
12-HETE	53.6656	23.73025	110.0985	55.4523	*
5oxoETE	12.0962	8.44525	11.9253	6.40004	-
5-HETE	3.77	2.66293	3.0749	1.34329	-

## Data Availability

Data is the part of the Ph.D. thesis of Ph.D. Marta Skórka-Majewicz. The data is available in the repository of the library of the Pomeranian Medical University in Szczecin.
